# Les urgences urologiques dans deux hôpitaux universitaires de Douala: une étude rétrospective (2016-2020)

**DOI:** 10.11604/pamj.2023.44.135.35954

**Published:** 2023-03-16

**Authors:** Frantz Guy Epoupa Ngalle, Landry Oriole Mbouche, Axel Stephane Nwaha Makon, Jean Cedrick Fouda, Junior Barthelemy Mekeme Mekeme, Armel Quentin Essomba, Jean-Jacques Nwatchap, Edouard Herve Moby Mpah

**Affiliations:** 1 Département de Chirurgie et Spécialités, Faculté de Médecine et des Sciences Biomédicales de l´Université de Yaoundé I, Yaoundé, Cameroun,; 2 Unité d´Urologie, Département de Chirurgie et Disciplines Affinitaires, Service de Chirurgie Général, Hôpital Général de Douala, Douala, Cameroun,; 3 Service d´Urologie, Hôpital Laquintinie de Douala, Douala, Cameroun,; 4 Département de Chirurgie et Spécialités, Faculté de Médecine et des Sciences Pharmaceutiques de l´Université de Douala, Douala, Cameroun

**Keywords:** Profil, urgences urologiques, clinique, traitement, Profile, urological emergencies, clinical, treatment

## Abstract

**Introduction:**

les urgences en urologie sont des situations urologiques critiques qui nécessitent une intervention rapide par un professionnel de santé qualifié en urologie. Cette étude a été menée dans le but de ressortir le profil des urgences urologiques reçues dans deux hôpitaux universitaires de la ville de Douala en appréciant leurs prises en charge en urgence.

**Méthodes:**

il s´agit d´une étude rétrospective portant sur les urgences urologiques dans deux hôpitaux de références de la ville de Douala que sont les hôpitaux Laquintinie et Général de Douala. Les dossiers ont été colligés durant une période de 5 ans (1^er^ janvier 2016 au 31 décembre 2020). Nous avons inclu toutes les consultations effectuées en urgence et reçues par le service des urgences ainsi que toutes les données cliniques et thérapeutiques venant du registre de garde durant la période d´étude. Nous avons exclu de notre étude toutes les urgences (consultations reçues pendant la période d´étude, non relevées dans le registre des urgences).

**Résultats:**

nous avons étudié 364 patients, l´âge moyen des patients était de 43 ± 8,34 ans. Quatre vingt-douze virgule cinquante huit pourcent (92,58%) (n=337) des patients étaient des hommes. Les principales urgences urologiques reçues étaient la rétention d´urine vésicale (45,05%, n=164), la colique néphrétique (15,33%, n=56) et l´hématurie (13,18%, n=48). Les principales étiologies des rétentions d´urine vésicale étaient les tumeurs prostatiques, la colique néphrétique était principalement d´origine lithiasique (96,45%, n=159) et l´hématurie était d´origine tumorale chez 68,75% (n=33) des patients. Sur le plan thérapeutique, les gestes effectués en urgence étaient le sondage vésical (39,01%, n=142), le traitement médical était associé à une surveillance (27,47%, n=100) et la cystostomie sus pubienne (10,71%, n=39).

**Conclusion:**

les rétentions aiguës d´urines vésicales sur tumeurs prostatiques constituent l´urgence urologique la plus fréquente dans les hôpitaux universitaires de la ville de Douala. Cela implique une prise en charge précoce et optimale des tumeurs prostatiques.

## Abstract (en)

**Introduction:**

urological emergencies are critical situations that require rapid response by a qualified urology health care professional. The purpose of this study was to highlight the profile of urological emergencies in two university hospitals in the city of Douala by assessing emergency management.

**Methods:**

we conducted a retrospective study of the urological emergencies in two reference hospitals in the city of Douala: the Laquintinie Hospital and the General Hospital. Files were collected over a period of 5 years (January 1^st^, 2016 to December 31^st^, 2020). All emergency consultations in the Emergency Unit as well as all clinical and therapeutic data from the on-call list during the study period were included. We excluded all emergencies (consultations during the study period) not recorded in the emergency registry.

**Results:**

we conducted a study of 364 patients with an average age of 43 ± 8.34 years; 92.58% (n=337) of patients were male. The most common urological emergencies included: urinary retention (45.05%, n=164), renal colic (15.33%, n=56) and haematuria (13.18%, n=48). The most common cause of urinary retention was prostate tumors, renal colic was mainly due to renal lithiasis (96.45%, n=159) and hematuria was due to tumor in 68.75% (n=33) of patients. Therapeutic management was based on urinary catheterization (39.01%, n=142), medical treatment was associated with monitoring (27.47%, n=100) and suprapubic cystostomy (10.71%, n=39).

**Conclusion:**

acute urinary retention due to prostate tumors is the most common urological emergency in the university hospitals in the city of Douala. Early and optimal management of prostate tumors is therefore essential.

## Introduction

Les urgences en urologie tout comme dans d´autres spécialités, évoquent une souffrance ou des situations de santé critiques nécessitant une prise en charge adéquate et immédiate par un professionnel de santé qualifié en urologie. Les urologues durant les permanences et les gardes se retrouvent souvent confrontés à de nombreuses urgences d´ordre traumatiques, infectieuses, obstructives, génito-scrotales et hématiques donc la rapidité de prise en charge conditionne le devenir du patient [1-3]. En France, une étude menée en 2019 révèle que sur 1257 patients ayant consulté dans une unité d´urgence urologique environ 45% des consultations ont nécessité une hospitalisation en urologie [4]. En Guinée, en 2009, les urgences urologiques représentaient 22% des urgences urologique au CHU de Conakry. Au Cameroun en 2012 selon Moby *et al*. les traumatismes des organes génitaux constituaient l´urgence andrologique la plus fréquente en milieu urbain. Dans notre étude, les urgences urologiques représentaient 51,50% des cas, suivis des infections urogénitales basses (23,71%). Ces données soulignent l´importance des urgences urologiques au Cameroun [5]. L´objectif de ce travail était de ressortir le profil épidémiologique, clinique et thérapeutique des urgences urologiques reçues dans deux hôpitaux universitaires de la ville de Douala au Cameroun.

## Méthodes

**Conception de l'étude:** il s´agissait d´une étude rétrospective descriptive.

**Contexte:** il s´agissait d´une étude des dossiers médicales sur les urgences urologiques colligées durant une période de 5 ans (1^er^ janvier 2016 au 31 décembre 2020). L´Hôpital Général de Douala (HGD) et L´Hôpital Laquintinie de Douala (HLD) étaient les deux hôpitaux de références sélectionnés pour cette étude.

**Population:** c´était un échantillonnage exhaustif des cas d´urgences urologiques enregistrés par le service des urgences de l´Hôpital Général de Douala (HGD) et celui de l´Hôpital Laquintinie de Douala (HLD). Dans notre étude, ont été inclues toutes les consultations et interventions chirurgicales effectuées en urgence et reçues par le service des urgences ainsi que toutes les données cliniques et thérapeutiques venant du registre de garde durant la période d´étude. Nous avions exclu de notre étude toutes les urgences (consultations et interventions chirurgicales) reçues pendant la période d´étude, non relevées dans le registre des urgences.

**Figure 1:**
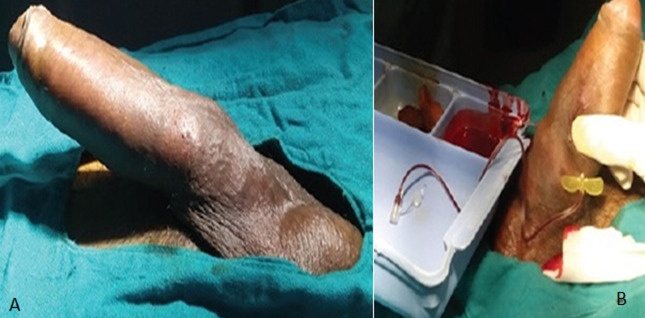
prise en charge d’un priapisme à bas débit

**Variables:** les variables étudiées étaient d´ordre épidémiologique (la fréquence, l´âge, le sexe), clinique (les motifs de consultation, et les pathologies diagnostiquées) et thérapeutique (la prise en charge en urgence) (Figure 1).

**Sources de données/mesures:** les données ont été collectées à travers les registres du service urgence et les dossiers médicaux stockés à la salle des archives du service d´urgence des deux hôpitaux. Toutes les données collectées ont été mesurées en fréquence et pourcentage.

**Biais:** pour réduire les risques de biais lié à la collecte des données, des personnels médicaux entrainés ont été sélectionnés pour cette tâche.

**Taille de l'étude:** nous avons inclus toutes les urgences urologiques reçues dans le service des urgences des deux hôpitaux pendant la période d´étude.

**Variables quantitatives:** les variables quantitatives ont été représentées en moyenne ± Ecart type (min-max).

**Analyses statistiques:** les données sociodémographiques, cliniques, et thérapeutiques ont été compilés et analysés sur le logiciel Excel. Les variables qualitatives ont été exprimées en effectif et pourcentage, dans des tableaux et texte.

## Résultats

**Présentation clinique:** entre le 1^er^ janvier 2016 et le 31 décembre 2020, 375 patients ont consultés pour une urgence urologique à l´Hôpital Général de Douala (HGD) et l´Hôpital Laquintinie de Douala (HLD) parmi lesquels 364 patients respectent nos critères d´inclusions. L´âge moyen des patients était de 43 ± 8,34 ans. Cinquante-un pourcent (51%) des patients avaient un âge supérieur à 60 ans avec des extrêmes d´âge allant de 1 à 91 ans. Quatre vingt douze virgule cinquante huit pourcent (92,58%) (n=337) des patients étaient des hommes.

**Figure 2:**
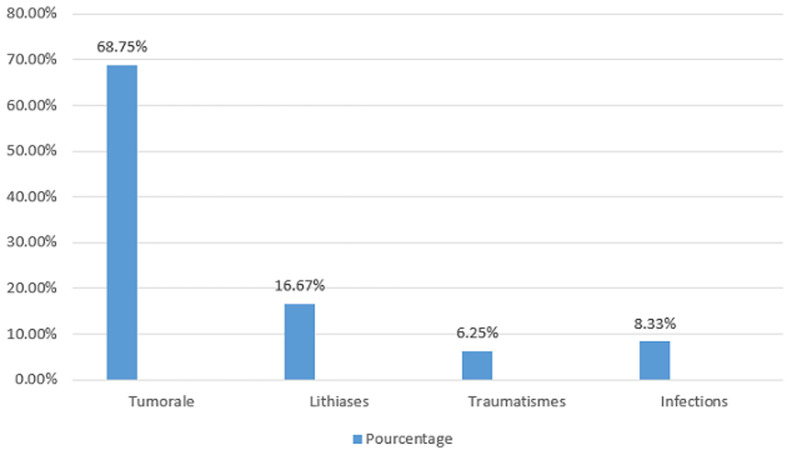
étiologies de l´hématurie

**Etiologies:** Les principales urgences urologiques reçues étaient la rétention d´urine vésicale (45,05%, n=164), les coliques néphrétiques (15,38%, n=56), les hématuries (13,18%, n=48), les torsions du cordon spermatique (8,24%, n=30) et la gangrène de Fournier (5,76%, n=21) (Tableau 1). Les principales étiologies des rétentions d´urine vésicale étaient les pathologies prostatiques; les hématuries étaient d´origine tumorale (68,75%, n=33), lithiasique (16,67%, n=8), infectieux (8,33%, n=4) et traumatique (6,25%, n=3) des cas (Figure 2). L´étiologie de la gangrène de Fournier était essentiellement urogénitale.

**Tableau 1:** distribution des urgences urologiques

Types d'urgence reçue à Douala	Nombre (n)	Pourcentage (%)
Rétention aigue d'urine	164	45,05
Colique néphrétique	56	15,38
Hématurie	48	13,18
Torsion du cordon spermatique	30	8,24
Gangrène de fournier	21	5,76
Pyélonéphrite aigue	21	5,76
Traumatisme des Organes Génitaux Externes	14	3,84
Rupture de l'urètre	6	1,64
Priapisme	3	0,82
Plaie hémorragique post-circoncision	1	0,27

**Prise en charge:** les gestes thérapeutiques effectués en urgence étaient le sondage vésical (39,01%, n=142), le traitement médical associé à une surveillance (27,47%, n=100), le lavage vésical associé à un décaillotage vésical (13,18%, n=48) la cystostomie sus pubienne (10,71%, n=39), la nécrosectomie en urgence (5,76%, n=21) et l´orchidopexie bilatérale (5,76%, n=21). Ces gestes effectués en urgence sont répertoriés dans le Tableau 2.

**Tableau 2:** distribution des actes thérapeutiques réalisés en urgence

Actes thérapeutiques réalisés	Nombre (n)	Pourcentage (%)
Sondage vésicale	142	39,01
Traitement médical + Surveillance	100	27,47
Lavage vésical + Décaillotage	48	13,18
Cystostomie sus pubienne	39	10,71
Orchidopexie bilatérale	21	5,76
Débridement(Nécrosectomie)	21	5,76
Exploration chirurgicale + Suture	12	3,29
Ponction sus pubienne à l'aiguille	12	3,29
Orchidectomie + Orchidopexie controlatérale	9	2,47
Urétroplastie	4	1,09
Ponction + Drainage des corps caverneux	3	0,82
Lithotripsie Vésicale	1	0,27

## Discussion

Dans notre étude plus de la moitié des patients étaient âgée de 60 ans et plus dont 92,58% d´entre eux furent des hommes. Les urgences urologiques retrouvées dans notre étude étaient la rétention aiguë d´urine (45,05%), la colique néphrétique (15,38%), l´hématurie (13,18%), la torsion du cordon spermatique (8,24%), les gangrènes de Fournier (5,73%), la pyélonéphrite aigue (5,73%) et les traumatismes uro-génitaux (5,48%). Toutes les tranches d´âge sont représentées dans notre étude, la majorité de nos patients (51%) avait plus de 60 ans. Ces résultats sont en concordance avec l´étude de Oumar Diallo *et al*. au Sénégal qui avaient aussi (51%) de patients d´un âge supérieur ou égal à 61 ans [6]. Cela peut être expliqué par le fait que les pathologies prostatiques surviennent généralement à partir de la cinquième décade, et que leur fréquence augmente avec l'âge [6]. Quatre vingt douze virgule cinquante huit pourcent (92,58%) des patients étaient des hommes. Cette prédominance masculine est en concordance avec Ouattara *et al*. au Burkina Faso [7]. Cette prédominance masculine peut être dû à une fréquence élevée d´urgence urétro-prostatique dans notre d´étude.

La rétention d´urine vésicale était l´urgence urologique la plus fréquente de notre série avec 45,05% des cas. Ce constat avait déjà été fait dans des pays africains notamment au Burkina Faso avec un pourcentage de 40,27% [7], 66,13% des cas au Sénégal [8] et 64% des cas au Togo [9]. Cela pourrait s´expliquer par des raisons socio-économiques et culturelles où le dépistage est rare et la plupart des patients consultent au stade de complication [6]. La fréquence importante de la colique néphrétique qui se positionne à la 2^e^ place parmi les urgences urologiques avec un pourcentage de 15,38%. Un constat similaire a été fait en France par Martin *et al*. qui enregistrait la colique néphrétique en 2^e^ place parmi les urgences urologiques (17,42%). Cependant au Cameroun ce taux varie d´une région à une autre. A Yaoundé, Owon´Abessolo *et al*. avaient retrouvé une fréquence de 10,5% (5^e^ place) [2]. Cela pourrait s´expliquer par le climat chaud, les habitudes alimentaires et les infections urinaires dans la ville d´étude. L´hématurie (13,18%) prend la 3^e^ place des urgences urologiques dans notre série. Le même constat a été fait au Burkina Faso en 2019 par Ouattara *et al*. qui les retrouvaient dans 21,09% des cas [7]. Cependant d´autres études placent l´hématurie menées en Mauritanie (5^e^ place), au Togo (4^e^ place) et au Sénégal (6^e^ place) [3,9,10]. Ceci pourrait s´expliquer par le fait que les études menées au Togo et en Mauritanie seraient réalisées en milieu hospitalier dans une zone urbaine. Les cas d´hématurie d´origine Bilharzienne sont traités en périphériques et donc ne parviennent pas au CHU et, au Sénégal; cela serait dû au fait que les hématuries intermittentes, minimes et sans caillotage vésicale n´étaient pas enregistrées comme urgence urologique. L´hématurie est la principale circonstance de découverte des tumeurs malignes de l´arbre urinaire [11].

La torsion du cordon spermatique représentait 8,24% dans notre série. Elle aboutissait à une orchidopexie bilatérale en urgence dans 5,76% des cas. Paradoxalement dans d´autres études camerounaises, le taux d´orchidectomie était plus élevé. Ceci peut se justifier par la prise en charge plus précoce de ses patients dans nos hôpitaux universitaires, les patients qui viennent ont plus de moyens ou sont assurés mais aussi le fait que les praticiens dans ses hôpitaux sont plus conservateurs [2,5]. Les urgences urologiques dues aux infections urogénitales sont dominées par les gangrènes de Fournier et la pyélonéphrite aigue qui représente chacun 5,76%. Cela est en désaccord avec le cas de Diabaté *et al*. au Sénégal où elles sont dominées par les orchiépididymites aigue [3]. Cela pourrait être dû à la différence dans les étiologies des 2 études. Les traumatismes uro-génitaux représentaient 5,48% dans notre étude dominée par les ruptures de l´urètre (1,64%). En corrélation avec les études faites par Diallo *et al*. et Owon´Abessolo *et al*. qui retrouvaient des résultats similaires [1,2]. Nous n´avons pas pu prendre tous les cas d´urgences urologiques reçus parce que certains dossiers médicaux avaient des informations incomplètes. Les dossiers médicaux des patients reçus en urgence urologique, qui étaient bien remplis ont été sélectionnés ce qui a facilité notre collecte de données.

## Conclusion

Les urgences urologiques dans la ville de Douala occupent une place importante dans la pratique urologique quotidienne. Elles sont dominées par les rétentions aiguë d´urines vésicales, la colique néphrétique et les hématuries. Elles concernent essentiellement les hommes âgés. Les urgences urologiques dues aux infections urogénitales sont dominées par les gangrènes de Fournier et la pyélonéphrite aiguë. Les traumatismes uro-génitaux sont dominés par les ruptures de l´urètre.

### Etat des connaissances sur le sujet

- *Les urologues sont très souvent confrontés à de nombreuses urgences urologiques donc la rapidité de prise en charge conditionne le devenir du patient;*
- *Les rétentions aiguës d´urines vésicales sont en prédominances en Afrique;*
- *La rareté de la torsion du cordon spermatique en Afrique comme urgence urologique*.

### Contribution de notre étude à la connaissance

- *Les urgences urologiques concernent essentiellement les hommes âgés;*
- *Les urgences urologiques dues aux infections urogénitales sont dominées par les gangrènes de Fournier et la pyélonéphrite aiguë;*
- *Les traumatismes uro-génitaux sont dominés par les ruptures de l´urètre*.

## Remerciements

Nous adressons nos remerciements à l'administration de l'Hôpital Général de Douala et de l´Hôpital Laquintinie de Douala.
